# Pitfalls of exome sequencing: a case study of the attribution of HABP2 rs7080536 in familial non-medullary thyroid cancer

**DOI:** 10.1038/s41525-017-0011-x

**Published:** 2017-03-28

**Authors:** Glenn S. Gerhard, Darrin V. Bann, James Broach, David Goldenberg

**Affiliations:** 1 Lewis Katz School of Medicine at Temple University, Philadelphia, PA 19140 USA; 2 Penn State College of Medicine, Hershey, PA 17033 USA

## Abstract

Next-generation sequencing using exome capture is a common approach used for analysis of familial cancer syndromes. Despite the development of robust computational algorithms, the accrued experience of analyzing exome data sets and published guidelines, the analytical process remains an ad hoc series of important decisions and interpretations that require significant oversight. Processes and tools used for sequence data generation have matured and are standardized to a significant degree. For the remainder of the analytical pipeline, however, the results can be highly dependent on the choices made and careful review of results. We used primary exome sequence data, generously provided by the corresponding author, from a family with highly penetrant familial non-medullary thyroid cancer reported to be caused by HABP2 rs7080536 to review the importance of several key steps in the application of exome sequencing for discovery of new familial cancer genes. Differences in allele frequencies across populations, probabilities of familial segregation, functional impact predictions, corroborating biological support, and inconsistent replication studies can play major roles in influencing interpretation of results. In the case of HABP2 rs7080536 and familial non-medullary thyroid cancer, these factors led to the conclusion of an association that most data and our re-analysis fail to support, although larger studies from diverse populations will be needed to definitively determine its role.

## Introduction

Next-generation sequencing using exome capture, commonly referred to as whole exome sequencing, has become a common approach used for the identification of single nucleotide variants (SNVs) associated with familial cancer predisposition syndromes. Exome sequencing targets essentially known annotated exons, while some versions of the library preparation reagents will also include coverage of untranslated regions and non-coding RNAs, and in some cases also allows the addition of custom contents. Exome sequencing has quickly emerged from its original application as a tool for gene discovery in research settings to an important diagnostic tool for clinical purposes,^1^ especially for diseases that may have significant genetic heterogeneity and require a multiplexed approach,^2^ such as inherited cancer syndromes.^3^ However, the entire exome sequencing process is highly complex with many uncontrollable variables contributing to both false positive and false negative results. Accordingly, the diagnostic rate for unselected patients undergoing exome sequencing is approximately 25%,^4, 5^ although much higher rates have recently been reported for certain conditions.^6^

For many cases in which exome sequencing has been used to identify variants associated with disease, the veracity of the association and the potential clinical significance of the variant are unclear, particularly when identified in a research setting. Even more worrisome is the assumption that such published research results often serve as de facto gold standards for translating to clinical practice. These concerns have been exemplified in a recent report by Gara *et al*. in which rs7080536 in the HABP2 gene was identified as the causative variant in a kindred with familial non-medullary thyroid cancer (FNMTC),^7^ a disorder for which no causal variants/genes have yet been identified.^8^ This result was brought into immediate question by several investigators,^9–13^ with a single positive association^14^ and a number of other contradictory follow-up studies described below. We used the data from Gara *et al.*,^7^ generously provided by the corresponding author, as a case study to discuss the aspects of exome sequencing that are particularly germane for the identification of genes underlying inherited disorders.

### Patient ascertainment and genetic model

Careful evaluation of the pedigree structure to generate hypotheses regarding the mode of inheritance of a presumed disease-causing allele is vitally important for exome sequencing. In the kindred identified by Gara *et al.*,^7^ the proband and five other family members were affected by non-medullary papillary thyroid cancer documented by thyroidectomy and pathological analysis of the thyroid tumor tissue. Given that disease was present in the proband and one brother out of seven total siblings, and was transmitted by the proband to one of two children and by the affected brother to all four of his children, an autosomal dominant mode of inheritance was postulated, consistent with available information on the familial transmission of non-medullary papillary thyroid cancer.^15^

Disease incidence also plays an important role in the design of studies. Gara *et al.* ^7^ regarded non-medullary papillary thyroid cancer as an uncommon disease. With an incidence of ~13.1/100,000 in the United States,^16^ and ~5% of cases familial,^17^ the estimated frequency of a presumed dominant acting familial mutant allele is about 1/300,000. However, the incidence of non-medullary papillary thyroid cancer appears to be increasing due to diagnosis of asymptomatic disease subsequent to improved diagnostic technology and increased surveillance, predominantly in young or middle-aged populations.^18^ In addition, a large meta-analysis of autopsy studies of thyroid cancer found that rate of incidental differentiated thyroid cancer based on partial thyroid gland histological analysis was 4.1% and of whole gland analysis was 11.2%.^19^ The relationship of this form of occult thyroid disease to highly penetrant FNMTC is not clear, but if the incidence of FNMTC is actually much higher then different study designs and methodologies should be used, as reported by others.^20^ Interestingly, three of the autopsy studies were from Japan, in which the rates of incidental differentiated thyroid cancer based on histological whole thyroid gland analysis were 15, 26, and 28%, much higher than most of the studies from European populations. HABP2 rs7080536 is not present in the 1000 genomes Japanese population, suggesting a low population allele frequency (AF) despite a potentially high rate of occult thyroid disease. Interpretation of association should therefore be based on accurate estimates of disease prevalence and allele frequencies.

### DNA sequencing

A number of different DNA sequencing platforms are now available for exome sequencing, although the field has generally coalesced around Illumina-based sequencing used by Gara *et al.* ^7^ and in several recent large series.^21–23^ Illumina-based sequencing appears to have a relatively lower error rate among current sequencing platforms,^24^ although the occurrence of such errors is often not acknowledged, and for which methods for correcting sequencing errors have been developed.^25^ Within the Illumina technology platform, the specific instrument used is also important. Deletions are more common than insertions using the HiSeq platform,^26^ while insertions occur more often than deletions when using the MiSeq platform.^27^

Errors may also be introduced during library construction from polymerase chain reaction (PCR) amplification. The presence of PCR-induced sequencing errors has led to the practice of confirming the results from exome sequencing using an orthogonal technology, especially for diagnostic applications.^28^ The pipeline used by Gara *et al.* ^7^ filtered low-quality sequence reads using criteria consistent with recent exome sequencing studies,^22, 23^ and then validated selected results using Sanger (automated fluorescence dideoxy) sequencing, considered the gold standard for exome sequencing validation.

### Data sharing and re-analysis

Though usually associated with genome-wide association studies, initial reports of genetic analysis often suffer from the “winner’s curse” phenomenon,^29^ with subsequent studies failing to replicate the initial finding. This has been the case for HABP2 rs7080536 and FNMTC, in which multiple reports found no association,^9–12, 20, 30–35^ and one found a positive association.^14^ Because of the multiple studies failing to replicate the association of HABP2 rs7080536 with FNMTC, we re-analyzed the raw sequencing data published by Gara *et al.*, whose corresponding author graciously provided complete access to the primary data, to determine whether we could obtain similar results*.* Data sharing is also extremely important in exome analysis because of differences in raw data, data processing, and analytical pipelines.

The raw FASTQ sequencing files were processed according to the Genome Analysis ToolKit (GATK) best practices pipeline,^36, 37^ a workflow similar to that used by Gara *et al.* who used an earlier version of GATK (v2.7.4 vs. v3.3.0) and a 2013 version of Annovar. Raw FASTQ files were obtained for patients II.2, II.3, III.1, III.2, III.3, III.4, III.5, III.6, III.7, III.8, IV.1, IV.3, IV.4, IV.6, and IV.7 based on the nomenclature used in the pedigree diagram^7^ and processed for analysis (Supplementary Methods). We found a high level of coverage, with an average of 94% of targeted bases covered to ≥10× across all patients (range 85.5 to 97.7%; data not shown). We identified a total of 230,495 variants across all of the family members. Gara *et al.* did not provide this number and it is not certain which individuals were included in their analyses.

We then utilized filtering criteria similar to the approach outlined by Gara *et al.* However, we did not include patient III.2, the unaffected daughter of the proband, due to the long latency period and high rate of occult disease associated with thyroid cancer, which makes it difficult to definitively classify this individual as unaffected. In addition, no individuals in Generation IV were used as they were likely too young for their disease status to be accurately ascertained. Exclusion of these individuals should not impact the identification of a causative variant but could decrease the number of potential candidates.

### Variant filtering by AF

Variant filtering also requires decisions regarding AF, genetic model, and expected disease prevalence. Gara *et al.* first filtered for variants at ≤1% AF in commonly used, publicly accessible population databases. This is a common initial step in an exome sequencing bioinformatics pipelines that permits a systematic evaluation of one or more genetic models using ethnicity-based stratification for AF and the exclusion of variants for which the AFs in available databases are not consistent with the genetic model.^22^ Thus, AF threshold data in reference databases are extremely important. The 1% AF selected by Gara *et al.* is a commonly used conservative initial threshold for a highly penetrant familial disorder with an autosomal dominant pattern of inheritance that will result in a significant reduction in numbers of variants without risking excluding a potential low-frequency causative variant.

The databases Gara *et al.* used for filtering included the 1000 Genomes Project^38^ and HapMap^39^ data (Table 1). The National Heart, Lung, and Blood Institute Grand Opportunity Exome Sequencing Project database (http://evs.gs.washington.edu/EVS/), which includes data on a set of DNA samples from 2203 unrelated African-American and 4300 unrelated European-American individuals analyzed by exome sequencing that is easily accessible, highly utilized for exome sequencing,^21–23^ and provides robust data on AFs (Table 1), was not used. In addition, the Exome Aggregation Consortium (ExAC) database,^40^ which includes data from 60,706 unrelated individuals and is becoming the de facto AF reference database, was also not utilized. Despite the strength of such large databases, they have significant limitations that may lead to erroneous attributions.^41^

**Table 1:** Allele frequencies for HABP2 rs7080536 in HapMap, 1000 genomes, Exome Variant Server and ExAC databases

Population	Allele A	Allele G	Genotype A|A	Genotype A|G	Genotype G|G
*HapMap* ^a^					
CSHL-HAPMAP:HapMap-CEU	0.018	0.982		0.036	0.964
CSHL-HAPMAP:HapMap-HCB	0.012	0.988		0.023	0.977
CSHL-HAPMAP:HAPMAP-MEX	0.031	0.969		0.061	0.939
CSHL-HAPMAP:HAPMAP-CHB	0.000	1.000		0.000	1.000
CSHL-HAPMAP:HapMap-JPT	0.012	0.988		0.024	0.976
CSHL-HAPMAP:HapMap-YRI	0.000	1.000		0.000	1.000
CSHL-HAPMAP:HAPMAP-TSI	0.023	0.977		0.047	0.953
CSHL-HAPMAP:HAPMAP-GIH	0.006	0.994		0.011	0.989
*1000 Genomes* ^b^					
1000GENOMES:phase_3_CDX		1.000			1.000
1000GENOMES:phase_3_JPT		1.000			1.000
1000GENOMES:phase_3_CEU	0.020	0.980		0.040	0.960
1000GENOMES:phase_3_PUR	0.019	0.981		0.038	0.962
1000GENOMES:phase_3_TSI	0.014	0.986		0.028	0.972
1000GENOMES:phase_3_YRI		1.000			1.000
1000GENOMES:phase_3_KHV		1.000			1.000
1000GENOMES:phase_3_SAS	0.004	0.996		0.008	0.992
1000GENOMES:phase_3_GIH	0.010	0.990		0.019	0.981
1000GENOMES:phase_3_AMR	0.014	0.986		0.029	0.971
1000GENOMES:phase_3_MXL	0.008	0.992		0.016	0.984
1000GENOMES:phase_3_EUR	0.027	0.973	0.002	0.050	0.948
01000GENOMES:phase_3_ALL	0.008	0.992	0.000	0.016	0.984
1000GENOMES:phase_3_PEL	0.006	0.994		0.012	0.988
1000GENOMES:phase_3_GBR	0.055	0.945	0.011	0.088	0.901
1000GENOMES:phase_3_MSL		1.000			1.000
1000GENOMES:phase_3_CHS		1.000			1.000
1000GENOMES:phase_3_AFR		1.000			1.000
1000GENOMES:phase_3_FIN	0.035	0.965		0.071	0.929
1000GENOMES:phase_3_BEB		1.000			1.000
1000GENOMES:phase_3_CHB		1.000			1.000
1000GENOMES:phase_3_STU		1.000			1.000
1000GENOMES:phase_3_IBS	0.014	0.986		0.028	0.972
1000GENOMES:phase_3_ASW		1.000			1.000
1000GENOMES:phase_3_ESN		1.000			1.000
1000GENOMES:phase_3_ASN		1.000			1.000
1000GENOMES:phase_3_ACB		1.000			1.000
1000GENOMES:phase_3_LWK		1.000			1.000
1000GENOMES:phase_3_GWD		1.000			1.000
1000GENOMES:phase_3_PJL	0.005	0.995		0.010	0.990
1000GENOMES:phase_3_ITU	0.005	0.995		0.010	0.990
1000GENOMES:phase_3_CLM	0.021	0.979		0.043	0.957
*Exome Variant Server* ^c^					
EVS EuropeanAmericanAlleleCount	0.038	0.961	0.001	0.075	0.923
EVS AfricanAmericanAlleleCount	0.007	0.993	0.000	0.013	0.987
*Exome Aggregation Consortium* ^d^					
European (non-Finnish)	0.033	0.967	0.001		
European (Finnish)	0.029	0.971	0.001		
South Asian	0.009	0.991	>0.001		
East Asian	0.000	1.000	0.000		
African	0.005	0.995	>0.001		
Latino	0.007	0.993	>0.001		
Other	0.030	0.970	0.000		

^a^ http://www.ncbi.nlm.nih.gov/projects/SNP/snp_ref.cgi?rs=rs7080536

^b^ http://browser.1000genomes.org/Homo_sapiens/Variation/Population?r=10:115347546-115348546;source=dbSNP;v=rs7080536;vdb=variation;vf=4906750

^c^ http://evs.gs.washington.edu/EVS/ServletManager?variantType=snp&popID=EuropeanAmerican&popID=AfricanAmerican&SNPSummary.x=29&SNPSummary.y=11&SNPSummary=Display+SNP+Summary

^d^ http://exac.broadinstitute.org/variant/10-115348046-G-A

Unfortunately, no details were provided in Gara *et al.* as to whether the global AFs from each database were used or whether the queries were population specific. The HABP2 rs7080536 AF in the HapMap database, obtained before the recent retiring of the database (which is now available only through archival download),^42^ indicated that the global AF across the eight populations, including its absence in two of them, was 1.25%. Similarly, the AF for HABP2 rs7080536 is 3.8% in the Exome Variant Server database, 3.3% in the ExAC database, and was 4–5% in a large genetic association study.^43^ The AF in several association studies cited by Gara *et al.* ranged from 2 to 5%.^44, 45^ The HABP2 rs7080536 global 1000 Genomes AF is <1%, although there is significant variation across populations. Indeed, the allele was not present in the Asian and African populations, but had a frequency of >1% in the European populations. Based on the HapMap and 1000 Genomes European AFs, as well as AFs reported in the reports cited by Gara *et al.*, the HABP2 rs7080536 should have been excluded by the initial filtering threshold of the analysis pipeline.

The HABP2 rs7080536 thus appears to have slipped under the AF filtering criterion threshold due to the large differences in AF across populations, a major factor when translating results from a small group of individuals to larger populations, especially across races/ethnicities.^41^ That non-European populations have much lower AFs for HABP2 rs7080536 likely explains the results of Gara *et al.* ^7^ that the frequency of HABP2 rs7080536 was 4.3% in The Cancer Genome Atlas (TCGA) samples, which were obtained from individuals largely of Caucasian/European ancestry,^20^ but was only 0.7% in a multiethnic population. What was thus interpreted as enrichment in individuals with thyroid cancer likely represents a discrepancy in germline AFs between populations consisting of different ethnic compositions, a classic pitfall in SNV interpretation.^41^

Because Gara *et al*. did not report the ethnicity of the index family, we sought to document that the ancestry of the family was from a population in which HABP2 rs7080536 is a common variant. We used iADMIX^46^ to estimate the ancestral composition for the family based on the HapMap v3 database.^47^ Our analysis revealed that the family was primarily of Northern and Western European Ancestry, with some similarity to the Toscani in Italia population (Supplementary Table 1). Therefore, the family appears to be from a Western European population where the expected AF for HABP2 rs7080536 is estimated to be at least 1%, if not several fold higher.

In our re-analysis of the Gara *et al.* data, we restricted the initial filtering to the 1000 Genomes database, omitting use of the HapMap data since the AF of HABP2 rs7080536 was >1%, as described above. We also conducted a separate analysis using a 1% threshold based on the 1000 Genomes CEU (Utah Residents with Northern and Western Ancestry) population. We identified 44,107 variants using the entire 1000 genomes data (Table 2) somewhat less than the 53,122 found by Gara *et al.*, which likely results from our exclusion of the individuals described above. Restricting our variant filtering pipeline to the 1000 Genomes CEU data resulted in 39,996 variants (Table 2).

**Table 2:** Re-analysis of Gara *et al.* exome data

Filtering step	Gara *et al.*	1000 G^a^	1000 G CEU^b^
(1) Variants identified	Not provided	230,495	230,495
(2) SNVs ≤1% in HapMap18^c^ and 1000 Genomes Databases	53,122	44,107	39,996
(3) SIFT score < 0.05 or not available	53,120	43,554	38,516
(4) In exonic region	3024	6556	4486
(5) Present in all three initial affected family members	20	709	600
(6) Nonsynonymous	4	388	284
(7) SNV/Indel is not present in unaffected/unrelated spouse	2	47	35
(8) Present in all seven affected family members based on screening of additional members	1	3	2

^a^ Global 1000 Genomes AF

^b^ AF in 1000 Genomes CEU (Utah Residents with Northern and Western Ancestry) population

^c^ The HapMap data was only used by Gara *et al.*

### Predicting variant effects on protein function

After AF-based filtering, many analytical pipelines filter for variants underlying missense substitutions that are predicted to cause a potentially functional amino acid change. Existing guidelines to predict potential deleteriousness have recommended that investigators “avoid considering any single method as definitive”.^48^ A variety of algorithms are available, including the computational SIFT (Sorting Intolerant from Tolerant) tool^49^ that Gara *et al*.^7^ used. Surprisingly, the often used PolyPhen algorithm,^50^ a workhorse application for exome sequencing,^22, 23^ was not used. We applied the criteria of a SIFT score < 0.05 or not available, which excluded 553 variants vs. the 2 excluded by Gara *et al.* The likelihood is low that only 2 variants out of 53,122 would have generated exclusionary SIFT scores. The use of other prediction algorithms may have highlighted discrepancies in the SIFT data.

### Familial segregation

Evidence of segregation of a variant with disease in families is also considered as significantly evidence of association. We determined how many variants were shared by the three initially affected family members analyzed by Gara *et al.* (Table 2). We found that just over 10% of the variants were shared by the three family members vs. 0.66% found by Gara *et al.* Our exclusion of individuals from the analysis likely contributes significantly to this difference. We also found over twice the percentage of shared variants predicted to be non-synonymous. These differences further highlight the need for data sharing given the potentially large effects upon results from seemingly reasonable and minor differences in approach.

The filtering pipeline used by Gara *et al*. identified a single variant, HABP2 rs7080536, shared by the seven affected family members, whereas we identified three variants using the entire 1000 Genomes data set in which HABP2 rs7080536 MAF is less than 1% and two variants using the 1000 Genomes CEU data (Table 2). Use of the 1000 Genomes CEU database excluded HABP2 rs7080536. Of the other two variants identified, one was absent from 1000 Genomes database because the population frequency was not determined, whereas it has an AF of >10% across all races/ethnicities in the ExAC database. The identification of this variant exposes another pitfall in exome pipelines; variants with absent data may be binned as low frequency rather than as no data producing another hidden cause of false positive interpretations. The other variant we identified, ZNF23 rs531705739, was also not present in the 1000 Genomes data-set but has an AF of only 0.0001773 in the ExAC database in the European population. The ZNF23 rs531705739 variant is predicted to result in a potentially damaging T40R amino acid substitution.

Reagents to prepare libraries for exome sequencing target exonic regions but may also capture reads from off-target non-exonic genomic regions, which may be used to identify high-quality variants.^51^ We initially limited our analysis to the target regions described by Gara *et al*.^7^ and then also accounted for variants outside of the exome target regions by using HaplotypeCaller to implement genome-wide joint variant calling. This strategy identified 2,048,043 genome-wide variants in the 15 individuals. Using a filtering strategy based on AF in both 1000 Genomes and ExAC resulted in the identification of the same single missense variant, ZNF23 rs531705739, but also 39 non-coding variants whose functional significance is not known and difficult to determine. Another important aspect of exome sequence analysis is that non-exonic variants may be found with unknown genetic significance.

### Corroborative biological support

In contrast to those without a likely genetic mechanism, exome sequencing studies often identify variants and genes for which the in silico support is strong, but for which little biological or clinical data exist. Studies are then undertaken to test hypotheses about the role of the variant in the disease process. Biological support for HABP2 rs7080536 generated by Gara *et al.* may have played a major role in the conclusion that it was the causative variant. The evidence presented was compelling from an in vitro perspective regarding a role for HABP2 rs7080536 in cancer biology, but not as a cancer-predisposing variant. For example, the variant was associated with increased HABP2 protein expression in tumor tissue from affected family members but no staining was found in normal adjacent thyroid tissue, suggesting that the protein may not be highly expressed in pre-malignant cells. Decreasing wild-type HABP2 expression through siRNA in two thyroid cell lines and HEK293 cells increased colony formation and cellular migration, while stable overexpression in the cell lines reduced colony formation and cellular migration. These data add further support to observations that HABP2 gene expression is dysregulated in various cancers, as recently suggested.^52^ However, similar to the HABP2 siRNA knockdown experiments, overexpression of the rs7080536 allele also increased colony formation, suggesting that rs7080536 is a loss-of-function allele as previously reported^53^ and not as a dominantly inherited gain-of-function allele. Balancing suggestive biological evidence with mixed genetic results further adds to the complexity of exome sequence analysis. ZNF23 has previously been implicated in human cancer,^54, 55^ although no mechanistic data yet links it specifically to thyroid cancer. Biological studies that do not adequately model the initial disease analyzed by exome sequencing should be interpreted very cautiously.

Many genes identified through exome sequencing are subsequently tested in animal models, which is considered a critical step for functional assessment and assigning of causality.^41^ Faithful replication of the disease/phenotype is generally considered strong evidence for validation. However, this may be problematic given the diversity of organisms used, commonly drosophila, zebrafish and mice, and relies on the relative degree of evolutionary conservation of particular proteins/pathways.^56^ No data from animal models were presented by Gara *et al*.^7^ Relying exclusively on in vitro data using transformed cells or cells of a different lineage carries multiple risks for over-interpretation.

Data are available in several publicly accessible databases that could have also been used to interrogate the potential biological relevance of HABP2 expression in thyroid cancer. We analyzed gene expression data for normal human tissues downloaded from the Uhlen’s Lab, GTEx, and Illumina Body Map databases within the European Molecular Biology Laboratory Gene Expression Atlas.^57^ We found that HABP2 was not expressed in the thyroid gland but was highly expressed in the liver (Supplementary Fig. 1), consistent with the observation of Gara *et al.* that normal thyroid tissue does not stain for HABP2,^7^ also corroborated experimentally.^20^ Despite its potentially compelling nature, such data can support, but not exclude, a cancer predisposition variant.

In light of the observations by Gara *et al.* that HABP2 was overexpressed in some thyroid cancers,^7^ we also used gene expression data from the TCGA^58^ to determine whether HABP2 overexpression is a common feature of thyroid cancer. We found that HABP2 was not expressed in >300 of the 505 thyroid tumors included in the TCGA data set and was expressed at only low to moderate levels in the remaining tumors (Supplementary Fig. 2), indicating that HABP2 overexpression is not a common feature of papillary thyroid cancer. No detectable RNA was found in the normal thyroid tissue or thyroid cancer in the Human Protein Database, although a low level of HABP2 protein was detected in normal thyroid.^52^ In contrast, ZNF23 was expressed at low levels by essentially all papillary thyroid cancers, consistent with its role as a transcription factor.

### Follow-up genetic studies

Replication of genetic results is perhaps the most highly regarded criterion for determining true associations. A variety of studies investigating the association of HABP2 rs7080536 with FNMTC and sporadic NMTC have been reported since the Gara *et al.* report. In addition to four letters responding to the initial report that did not find an association,^9–12^ no associations were found in subsequent populations from the United Kingdom,^30^ the United States,^20^ Saudi Arabia,^31^ Colombia,^32^ Spain,^33^ Italy,^34^ or Australia.^35^ Zhang and Xing identified the HABP2 rs7080536 variant in 4 of 29 (13.8%) of unrelated FNMTC kindreds.^14^ However, no statistical assessment was provided to determine whether this observation was different from that expected from the population frequency of the HABP2 G534E allele. Given the prevalence of HABP2 rs7080536 in the general population, Carvajal-Carmona *et al.* ^59^ have pointed out that there is “high probability (>10%) that HABP2 G534E will be present in 4 out of 29 families by chance”.^59^ Applying the Fisher’s exact test of proportions indicates that there is less than a 5% chance that a 1/29 (a 1/58 AF) proportion is different than 4/29. Due to differences in populations, study designs, and other factors, careful evaluation of replication results is warranted.

### Summary

Exome sequencing has become an invaluable tool for identifying variants associated with familial conditions. However, the complexity of the entire analytical and validation process requires rigorous application and interpretation of approaches and results. Identification of the HABP2 rs7080536 common variant as candidate for FNMTC was based largely on differences in allele frequencies across populations, familial segregation within a single pedigree, and mechanistic biological support. Follow-up studies have largely failed to replicate the association and application of stricter criteria in a re-analysis of the shared primary data identified for a rare missense variant that also segregated with disease. However, as recently proposed,^34^ larger studies from populations with low HABP2 rs7080536 allele frequencies will be needed to definitively assess its role in FNMTC. Careful attention to the key steps in exome analysis is important to maximize accurate interpretation of results.

## Electronic supplementary material

Supplementary Material
