# Supplementary material for: Pitfalls of exome sequencing: a case study of the attribution of HABP2 rs7080536 in familial non-medullary thyroid cancer
Source: NPJ Genom Med. 2017 Mar 28;2:8. doi: 10.1038/s41525-017-0011-x (PMC5584869; doi:10.1038/s41525-017-0011-x)
Supplement: Supplementary file 1 — Supplementary Material [file 41525_2017_11_MOESM1_ESM.docx]

Supplementary Materials

Pitfalls of exome sequencing: a case study of the attribution of HABP2 rs7080536 in familial non-medullary thyroid cancer.

Gerhard, et al.

1. Supplementary Methods

2. Supplementary Table 1: Estimated ancestral admixtures of the family reported by Gara et al. based on eight populations from the HapMap v3 database.

3. Supplementary Figure 1: HABP2 expression in normal tissue.

4. Supplementary Figure 2: HABP2 expression in thyroid carcinoma.

**Supplementary Methods**

Raw reads were aligned to the hg19 human reference genome using Burrows-Wheeler Aligner v0.7.12,33 and converted to the .bam file format and sorted using Samtools v0.1.19.34 PCR duplicates were removed using Picard v1.102.35 Indel realignment and base quality score recalibration was accomplished using the RealignerTargetCreator, IndelRealigner, and BaseRecalibrator tools in GATK v3.3.0.36 Joint variant calling across regions targeted by the Agilent 71 Mb whole exome panel ± 100 bp was conducted for all individuals using GATK HaplotypeCaller. Variant quality score recalibration was conducted using the VariantRecalibrator and ApplyRecalibration tools in GATK v3.3.0.36 Identified variants were annotated using Variant Effect Predictor v8338 and loaded into GEMINI v0.18.339 for analysis and filtering. For genome-wide analysis, variant calling using the HaplotypeCaller tool in GATK was not limited to exonic regions, allowing for the identification of high-quality variants in off-target reads.

| Supplementary Table 1. Estimated ancestral admixtures of the family reported by Gara *et al.* based on eight populations from the HapMap v3 database. | | | | | | | | |
| --- | --- | --- | --- | --- | --- | --- | --- | --- |
| Individual | YRI | CHB | CHD | TSI | MKK | LWK | CEU | JPT |
| II.2 | 0 | 0 | 0 | 0.293 | 0 | 0 | 0.707 | 0 |
| II.3 | 0 | 0 | 0 | 0.292 | 0 | 0 | 0.708 | 0 |
| III.1 | 0 | 0 | 0 | 0.349 | 0 | 0 | 0.651 | 0 |
| III.2 | 0 | 0 | 0 | 0.369 | 0 | 0 | 0.631 | 0 |
| III.3 | 0 | 0 | 0 | 0.271 | 0 | 0 | 0.729 | 0 |
| III.4 | 0 | 0 | 0 | 0.311 | 0 | 0 | 0.689 | 0 |
| III.5 | 0 | 0 | 0 | 0.231 | 0 | 0 | 0.740 | 0.029 |
| III.6 | 0 | 0 | 0 | 0.302 | 0 | 0 | 0.698 | 0 |
| III.7 | 0 | 0 | 0 | 0.345 | 0 | 0 | 0.655 | 0 |
| III.8 | 0 | 0 | 0 | 0.305 | 0 | 0 | 0.695 | 0 |
| YRI – Yoruba in Ibadan, Nigeria; CHB – Han Chinese in Beijing, China; CHD – Chinese in Metropolitan Denver, CO; TSI – Toscani in Italia; MKK – Maasai in Kinawa, Kenya; LWK – Luhya in Webuye, Kenya; CEU – Utah residents with Northern and Western European Ancestry from the CEPH collection; JPT – Japanese in Tokyo, Japan | | | | | | | | |


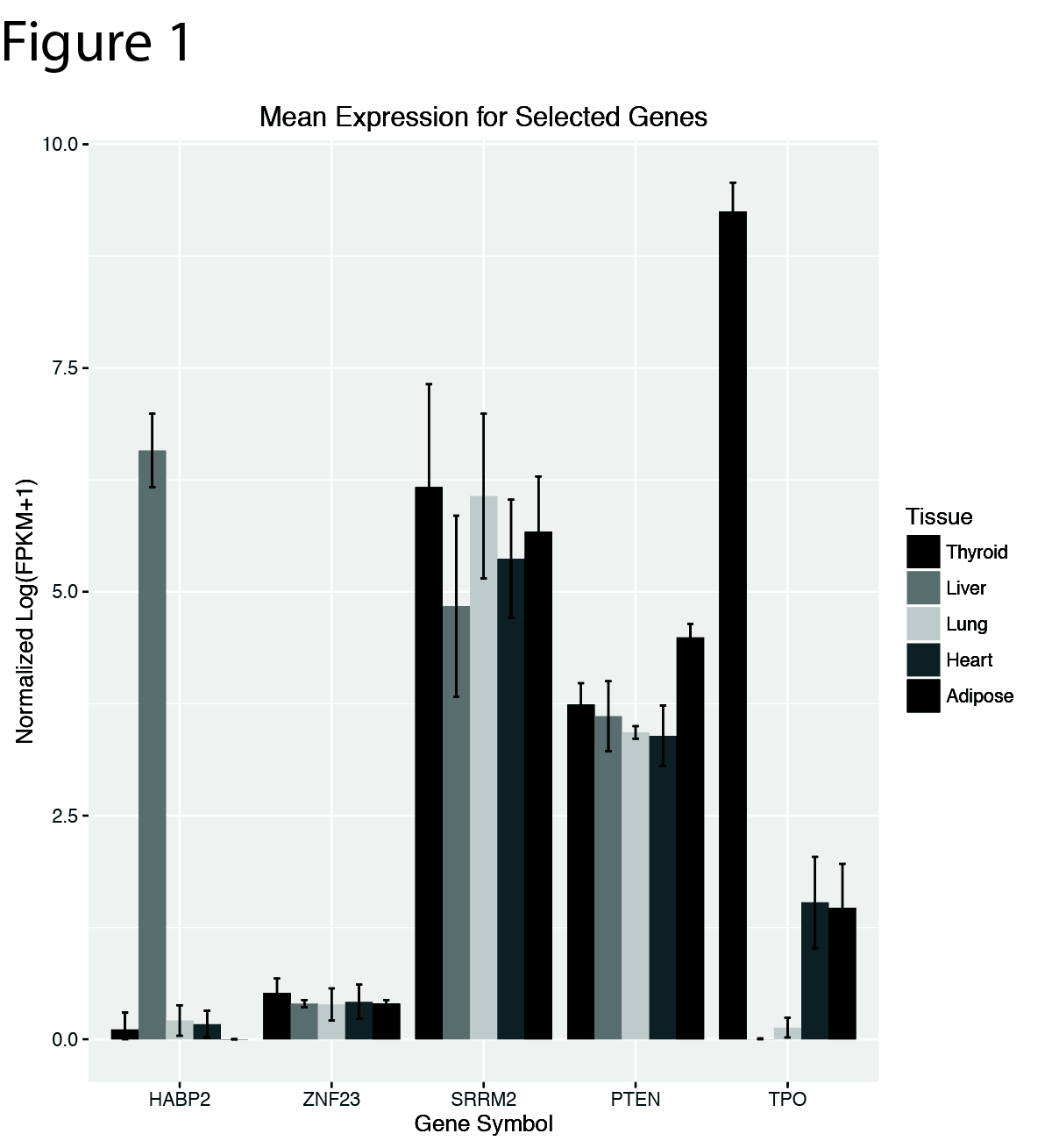


Figure 1. **HABP2 expression in normal tissue**. RNA sequencing data files were downloaded from the Uhlen’s Lab, GTEx, and Illumina Body Map datasets in the EMBL Gene Expression Atlas. To compare results between databases, raw FPKM RNA sequencing (RNA-seq) expression values were normalized by the following method: 1) genes where expression was equal to 0 across all tissues were removed; 2) raw FPKM values were log-transformed by Log_2_(FPKM+1); and 3) log-transformed values were quantile normalized and plotted using the preprocessCore and ggplot2 packages for R v3.2.2.[^59^](#_ENREF_59)^,^[^60^](#_ENREF_60) Gene expression values were calculated as the mean value across all three datasets plus or minus the standard deviation, and plotted using the ggplot2 package for R v3.2.2.[^60^](#_ENREF_60). PTEN, a well-characterized tumor suppressor associated with the familial tumor predisposition Cowden syndrome that includes papillary thyroid cancer as part of the clinical spectrum, was moderately expressed across a variety of tissues, and was expressed at a significantly higher level than HABP2 in the thyroid gland. As expected, thyroid peroxidase (TPO), was highly expressed in the thyroid gland. ZNF23 was expressed at higher levels than HABP2 in most tissues, while SRRM2 was broadly expressed at much higher levels than HABP2.


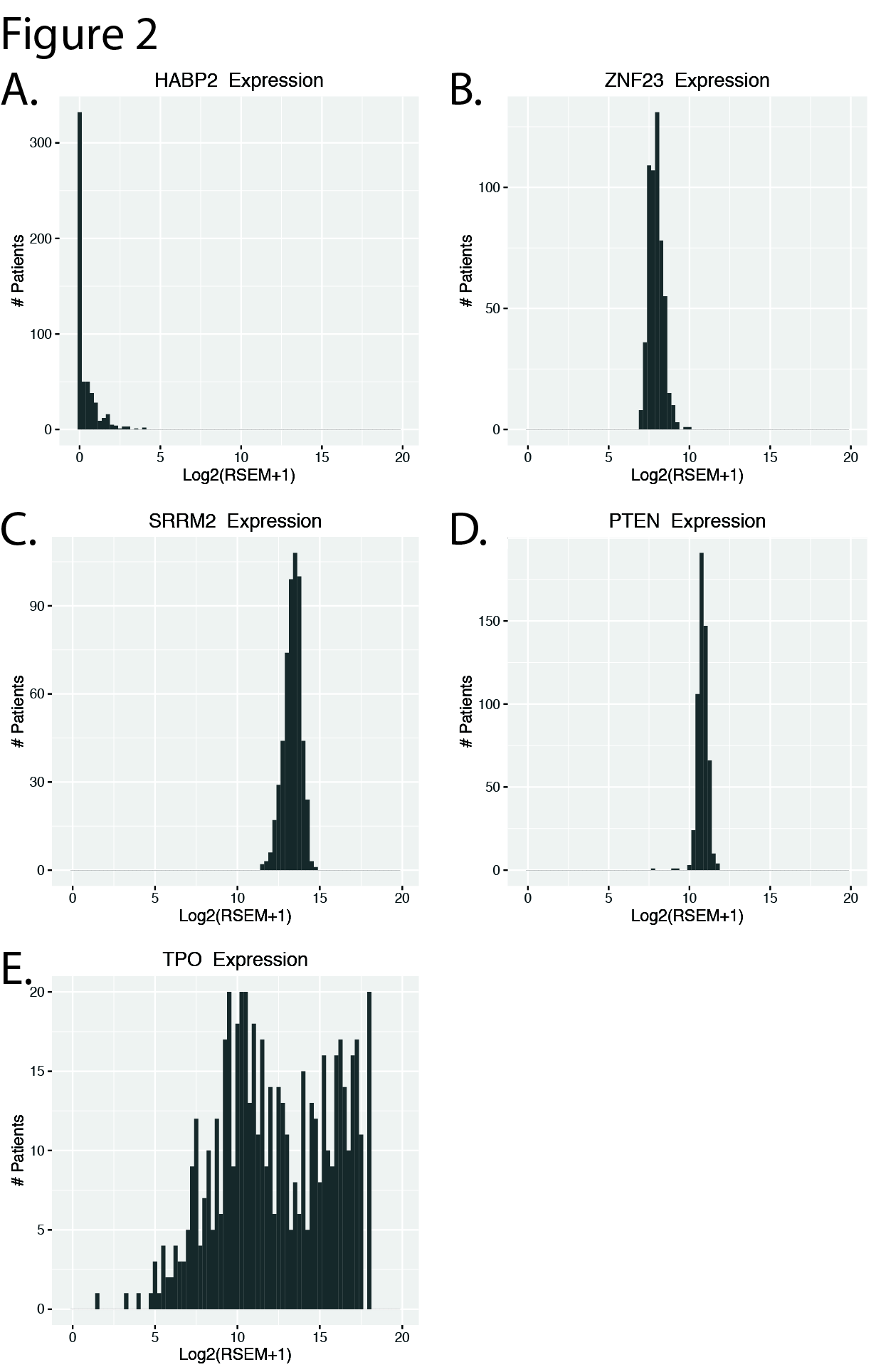


Figure 2. **HABP2 expression in thyroid carcinoma.** Histograms show top quintile-normalized RSEM RNAseq gene expression data for (A) HABP2, (B) ZNF23, (C) SRRM2, (D) PTEN, and (E) TPO from all 505 thyroid carcinoma cases analyzed by the Cancer Genome Atlas Research Network (TCGA). Histograms were generated using the ggplot2 package for R v3.2.2. HABP2 expression was undetectable in a majority of the tumors in the TCGA dataset.
